# Investigation of Modified Auxetic Structures from Rigid Rotating Squares

**DOI:** 10.3390/ma15082848

**Published:** 2022-04-13

**Authors:** Julian Plewa, Małgorzata Płońska, Paweł Lis

**Affiliations:** Faculty of Science and Technology, Institute of Materials Engineering, University of Silesia in Katowice, 75 Pułku Piechoty Str. 1a, 41-500 Chorzów, Poland; malgorzata.plonska@us.edu.pl (M.P.); pawlis4708@gmail.com (P.L.)

**Keywords:** auxetic structures, metamaterials, negative Poisson’s ratio (NPR)

## Abstract

Auxetic structures exhibit unusual changes in size, expanding laterally upon stretching instead of contracting. This paper presents this effect in a failsafe mode in structures made of rigid squares. We applied the concept of auxetic structures made of rigid rotating squares (from Grima and Evans) and offer a novel solution for connecting them. By introducing axes of rotation on the surface of the squares, a reliable working system is obtained, free from stress, in which the squares can come into contact with each other and completely cover the surface of the structure, or, in the open position, form regularly arranged pores. Herein, we present a new 2D auxetic metamaterial that is mathematically generated based on a theoretical relationship of the angle between the edges of a square and the position of the axis of rotation. Physical models were generated in the form of a planar structure and in the form of a circular closed structure. Such physical models confirmed our initial considerations and the geometrical relationships, offering new application possibilities. The novel structure that was designed and manufactured for the purpose of the paper can be considered as a new proposal in the market of auxetic materials.

## 1. Introduction

Mechanical metamaterials are a group of materials whose properties are controlled by their topology. These are mechanical properties in which the elastic properties (bulk modulus, shear modulus, Young’s modulus and Poisson’s ratio) can have different values than in ordinary objects. These systems are said to have abnormal properties under tensile or compressive stress.

Two groups of mechanical metamaterials can be distinguished, namely auxetic metamaterials (with a negative Poisson’s ratio—NPR) and non-auxetic metamaterials (with a positive Poisson’s ratio) [1]. Both of these groups have a significant potential for application, which, though not without difficulty, is becoming commercialized, e.g., their multifunctional applications in energy storage, biomedicine, acoustics, photonics and thermal management, architecture, mechanical engineering [2,3] and mechatronics—in sensors and actuators [4].

Among auxetics, which are special geometric constructions of regularly arranged elements (unit cells) forming a continuous system subjected to a force field, two basic systems are generally distinguished: the lattice structure and other structural arrangements [5]. In these systems, unit cells (internal auxetic units) are in a particular configuration, among which, two play a significant role. These are re-entrant honeycomb cells [6] and rotating rigid regular geometric shapes [7]. These units usually consist of beams, trusses or shells (plates), with varying degrees of arrangement in space to form complex base objects of auxetic structures.

Honeycomb cells have become the basis for a large number of solutions, complex units of re-entrant structural cells, some of which have found practical applications. A particularly interesting group of auxetics was produced from polymers, which includes re-entrant foams [8].

From the point of view of the deformation mechanism occurring at the interaction of the auxetic material and mechanical energy [9], auxetic metamaterials are usually classified as re-entrant [10,11], chiral [12,13], rotating [14,15] and laminated with a hierarchical structure [16,17]. Such cellular structures are designed using complex algorithms and computer procedures [10,18,19] and manufactured using a 3D printing technique. This technique was adopted from additive manufacturing and also gives the possibility to create moving parts [20].

The observed progress in the research and development of mechanical metamaterials is encouraging for examining such structures and confirming their extraordinary properties. Therefore, the aim of this work was to produce auxetic structures and to find the mathematical relations that occur in them. Two-dimensional structures of interconnected moving rigid elements are taken as the starting point in this work.

At this point, it seems fitting to quote the creators of ‘rotating squares’ [11]: “[...] the rotating squares mechanism has established itself as one of the leading models which can explain auxeticity in a number of materials, where it has stood the test of time as a robust and highly applicable model”.

Already considered classical, the structure of rotating squares [7], in which the squares are connected at the vertices and can rotate freely when a tensile force is applied, expands both upwards and downwards. This auxetic behavior can also be observed in structures composed of rectangles, triangles and parallelograms. Models based on this invention have one major disadvantage: the vertex connections of the squares are unstable because they exhibit high-stress states—the so-called critical stresses generated at the joints [21]. Their properties depend on both the structure and the type of material used.

In the presented paper, as a novelty in creating auxetic structures, a fundamental improvement of the design has been proposed, consisting of the stable connection of the rotating unit cells. A novel solution to connecting square frames of the structure was used in the form of rotation axes on their surface at a preset distance from the edge of the square cell. These axes of rotation located on the surface of the square rigid unit cells close to their corners allow for easy rotational movement with almost no friction. Favorable conditions have thus created the realization of the auxetic structure’s hinge mechanism, consisting of the easy and reversible twisting of the elementary cells concerning each other. The easy assembly of such improved structures consists of connecting square cells with holes near the corners by pin or bolt type elements.

Considering the structure of the rotating squares, and proposed moving the axis of rotation from the vertices to the surfaces of the squares, allowed obtaining two-dimensional auxetic structures that do not become damaged and that have properties that do not depend on the type of rigid material used. The elastic modulus of the material is, therefore, not applicable in this case, leaving only the Poisson’s ratio for this structure.

## 2. Designing Auxetic Structures Made of Rigid Elements in the Form of Squares

Structures built from polygons, based on various connecting elements have been described in a number of papers. In work [22], the authors used thin, flexible flat ligaments attached to the squares from underneath, obtaining a physical prototype with auxetic properties. Three-layer hinges (upper and lower layers of rigid material, with an embedded flexible part in between) were used in interconnected rigid and moving parts [23]. Other solutions involve forming a neck-like polymer part to connect the squares [24]. Such connections can also be found in perforated structures (stiffened elliptical perforated structures, e.g., [25]).

Connecting the elements with pivots is also a possible alternative [26], and the extensive literature of the subject provides further examples of connecting the squares and creating auxetic structures on this basis. In order to rotate rigid square-shaped units and to build hierarchical structures, adjacent units were connected on the edges between the vertices. Holes made in the four vertices were used for pin joints [27]. This solution relies on previous work [28], which showed a set of simple cuts to obtain a wide range of desired shapes and patterns, including hinges consisting of partially cut units. Such solutions lead to stress concentration at the hinges because, as reported in [28,29], the hinge design represents a trade-off between hinge failure and hinge stiffness.

Studies of structures equipped with such hinges allow determining elastic properties of auxetic structures. The first step is always the geometric analysis, which is carried out for the idealized system in which hinges are assumed to be point contacts of the square corners. Examples of such analyses can be found in, e.g., [7,22].

### 2.1. Geometrical Relationships of the Auxetic Structure Made from Rigid Squares

**Figure 1:**
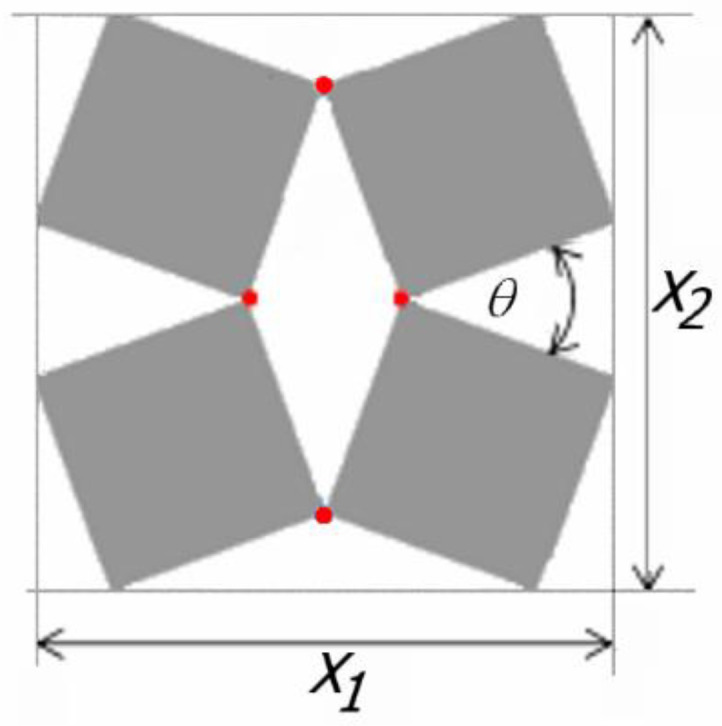
Model of rotating squares with marked hinges similar to Grima and Evans [7].

A detailed analysis of the relations between rigid squares connected with each other at the vertices by hinges was presented in [7] (shown in Figure 1).

It is demonstrated that, for a set of four connected squares (2 × 2), the size of the auxetic structure is expressed by the following formula [7]

(1) $$X_{1}=X_{2}=2l\left(cos\frac{\theta }{2}+sin\frac{\theta }{2}\right)$$

**Figure 2:**
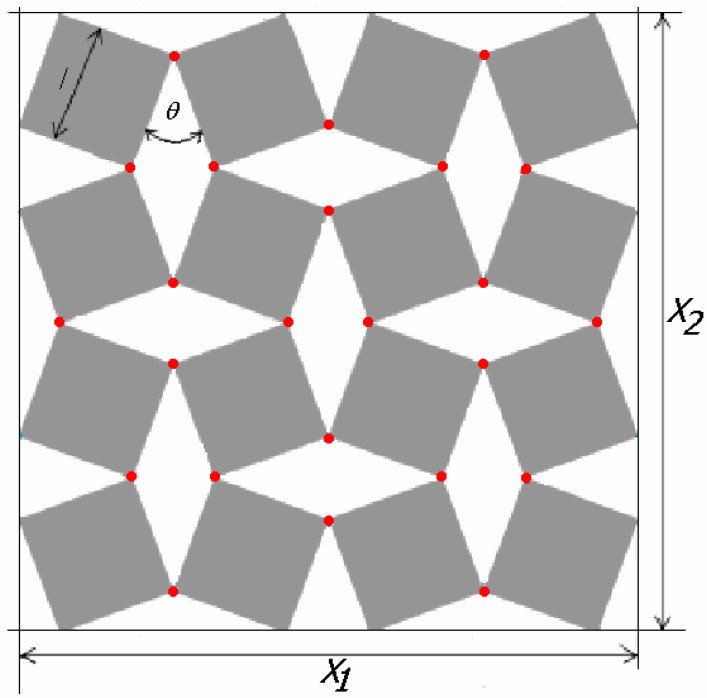
The rotating squares structure in a partially open position with marked hinges a × x similar to Grima and Evans [7].

This relationship is modified when the set is enlarged to sixteen squares (4 × 4), in which case the dimensions of the structure will depend on the direction (*X*_1_ ≠ *X*_2_) (Figure 2) [22]. Of particular importance in these considerations is the angle *θ*, varying from zero closed structure) to 90° (open structure).

Proposing a new approach to the creation of auxetic metamaterials based on the configurations of rigid units, a modification is introduced involving the creation of permanent and mobile connections. If the axes of rotation of the squares are moved from the vertices to their surfaces and placed on the diagonals, the above geometrical situation will not change, although the squares will then overlap.

The above diagram emphasizes that, in such an auxetic construction, the squares are subject to not only rotational motion on the axes but also translation, as are the axes of rotation. It follows that the expansion of the structure from the closed to the open position involves both rotational motion and linear displacement.

**Figure 3:**
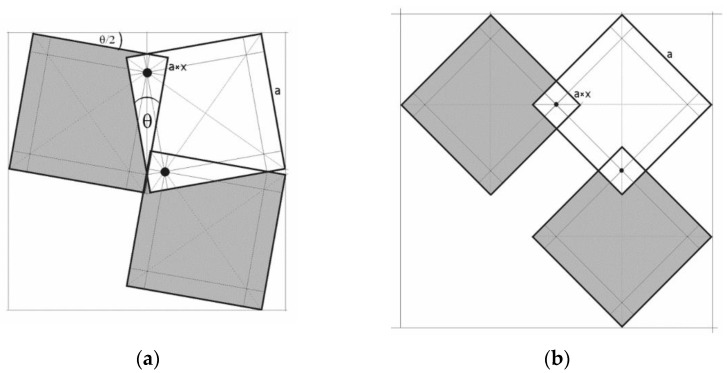
Connection of the rotating squares with the axes on their surface for the closed position (**a**) and for the open position (**b**).

Figure 3 shows the axis of rotation, which lies at a distance a × x from the edge of the square and at a distance a√2 × x from its corner. With such a designation, the following relation occurs:

(2) $$tan\frac{\theta }{2}=\frac{1+\surd (1-4x)}{1-\surd (1-4x)}$$

**Figure 4:**
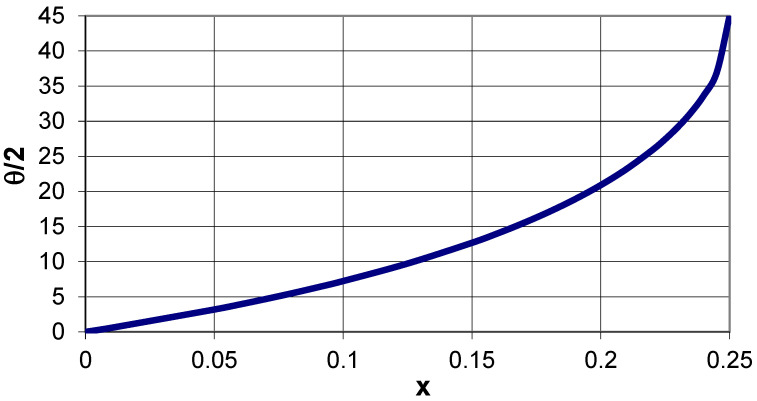
The theoretical relationship between the internal angle of the structure and the parameter x.

The internal angle *θ* between two edges of the squares corresponds to the closed structure, i.e., the position where two other vertices of adjacent squares meet. As a result of rotation around the axis, the structure will become open, and the angle created between the edges of the squares will be equal to 90°. Figure 4 shows the theoretical variation of the internal angle *θ*/2 as a function of the parameter x. It needs to be added here that Equation (2) represents a useful relation that enables building an auxetic structure in the closed position for a given value of parameter x. Without being aware of this relation, it is difficult to draw such a geometrical structure. For a value of x = 0.25, the theta angle takes the value 0.5*θ* = 45°, which can be treated as a borderline case in which all the squares are arranged so densely that they block one another, and no rotation is possible.

This structure can be described as a new version of the well-known two-dimensional ‘rotating squares’ model. Such improvement can be achieved by introducing additional elements in the form of axes of rotation within the planes of the squares, which causes the system to become a two-dimensional auxetic when stretched. In the closed position, the two edges are in contact. However, in the open position, squares of area (2a × x)^2^ overlap. From a geometrical point of view, the mechanism shown is a consequence of the opening of squares with one edge in contact with and anchored on the axis of rotation, which leads to an alignment of the angles between the edges in the fully opened position.

### 2.2. Poisson’s Ratio of the Auxetic Structure

The pairs of connected squares shown above can be further combined to form larger structures with a Poisson’s ratio corresponding to the difference in size in the closed and open positions. When twisted around the axis of rotation, the structure expands both horizontally and vertically and reaches its maximum size. Both *X*_1_ values and *X*_2_ values increase uniformly.

**Figure 5:**
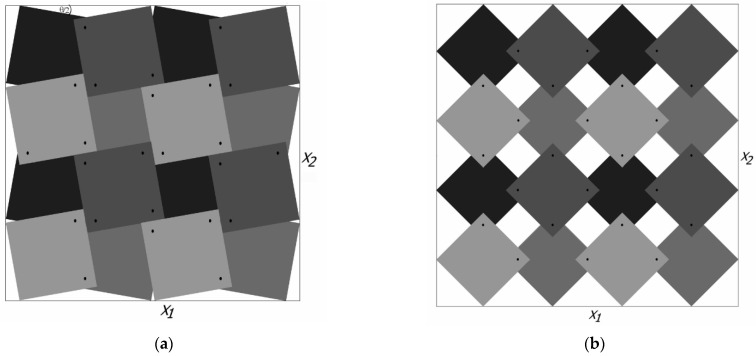
Auxetic structure 4 × 4 in the closed (**a**) and open (**b**) position for x = 1/7.

In a structure subjected to a force acting in a vertical or horizontal direction, the position of each square changes relative to the plane of the structure’s surface. This results in the opening up of the entire structure leading to increased dimensions in both the horizontal and vertical directions as well as the NPR effect. Analyzing the geometrical relationships within the structure *X*_1_ and *X*_2_ can be calculated. The horizontal and vertical size differences for open and closed positions are equal to each other, leading to a constant Poisson’s ratio value of −1. In the presented structure (Figure 5), the determined Poisson’s ratio does not depend on the mechanical properties of the rigid squares, and the tensile force is small and depends only on the friction on the squares’ surface.

### 2.3. Pore Surface of an Open Auxetic Structure

**Figure 6:**
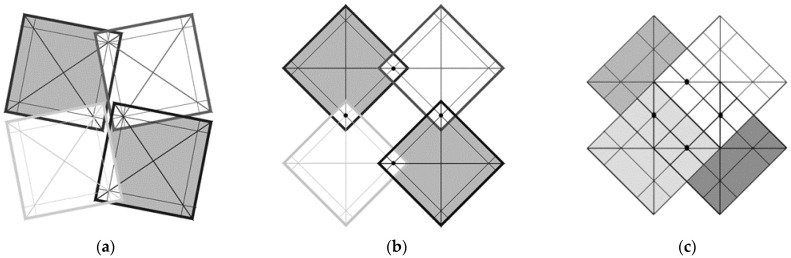
Auxetic structure 2 × 2 in partially open (**a**) and open positions (**b**) for x = 0.11, $\frac{\theta }{2}$ = 7.2° (according to the Equation (2)) together with the structure for x = 0.25 (**c**).

The presented at Figure 6 auxetic structure of the type 4 × 4 is characterized by the fact that for the closed position *X*_1_ = *X*_2_, while for the open position *X*_1_ = *X*_2_. However, the change in size in the horizontal direction and in the vertical direction is the same. While in the closed position, the structure is ‘tight’—it does not have any pores; in the open position, square-shaped pores of side length (a−a × 4x) are formed. This indicates that the further away from the corner of the square the axis of rotation is, the smaller is the pore size.

For a parameter value x = 0.25, the pores disappear, which means that the squares cannot rotate, and their surfaces overlap to a greater extent. In the case where x = 0.25, the structure becomes immobile because the touching edges of adjacent squares block their rotation. While in the closed position (Figure 6a), the surface is entirely covered by the squares, and, in the open position (Figure 6b), square-shaped pores are formed. The theoretical change in the pore surface can be calculated as the square area of (a−a × x)^2^ (Figure 6c).

**Figure 7:**
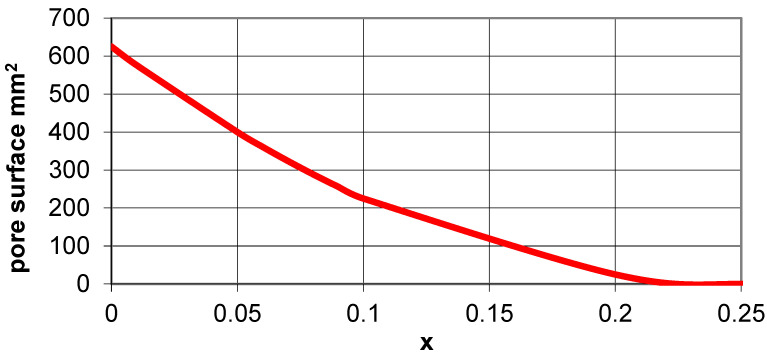
Change in the pore surface size in an open auxetic structure as a function of parameter x.

Figure 7 demonstrates the relationship for the change in pore surface in a 2 × 2 structure (for a = 25 mm). It shows that, with the increase of the parameter x—the shift of the axis of rotation to the center of the square—the surface area of the pores decreases very strongly, i.e., the structure increasingly covers the surface and becomes less mobile. The pores are empty spaces that can be used, for example, in adjustable filtration systems.

## 3. Physical Structure Models

**Figure 8:**
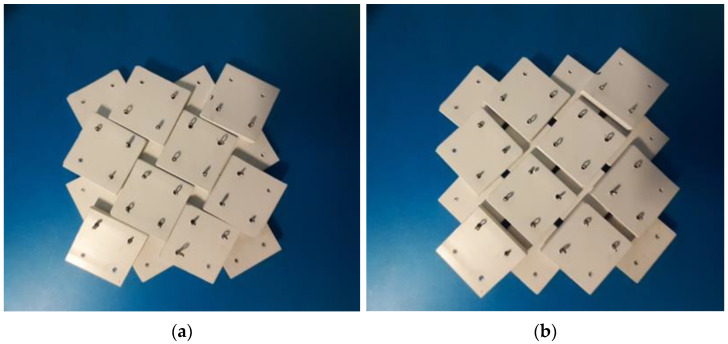
Photographs of auxetic structures 4 × 4 for x = 0.21 in the closed (**a**) and open positions (**b**).

In the course of the research, a considerable amount of modeling work was carried out on the system of rotating squares with rotation axes on the surface of the squares. The applied concept was visualized with the use of physical models (Figure 8). Initially, the structures were tested for different positions of the axis of rotation.

Using 2 mm thick rigid polymer plates in the form of squares of a = 23.5 mm, models were made for x = 5/23.5.

One can see that, in the closed position, the structure completely covers the surface, while in the open position, pores are formed with their size closely related to the parameter x. The considerable displacement of the rotation axis—the distance from the edge equal to 5mm (x = 5/23.5) of the 23.5 mm × 23.5 mm squares—makes the pores small and, considering the size of the rotation axis (2 mm diameter holes), the surface of one pore is less than 3.5 × 3.5 mm^2^. Very little force is required to change the position between the closed and open states, even though very primitive rotation axes in the form of wire from metal split pins were used.

The physical model is easily stretched and assumes both open and closed positions. The rigid squares connected in the rotation axes overlap both in the closed and open positions. In the closed position, however, parts of the edges of adjacent squares come into contact with each other.

**Figure 9:**
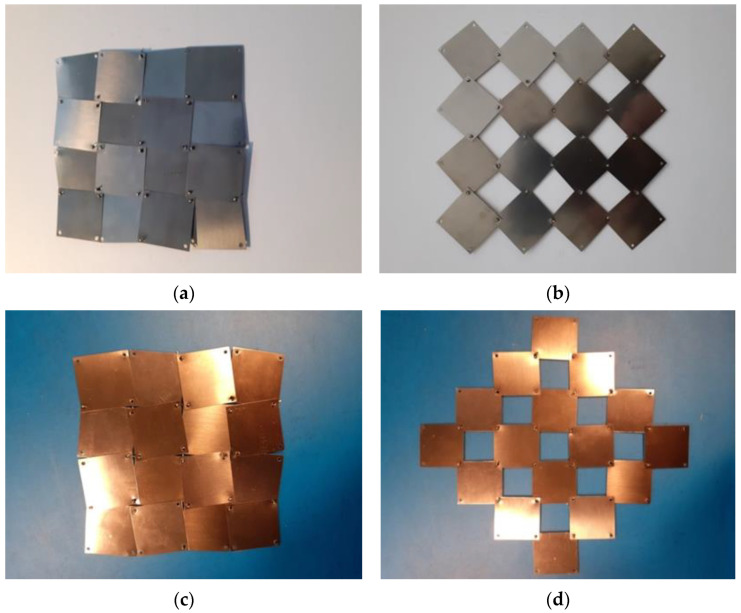
Auxetic structures 4 × 4 from thin steel sheets (**a**) and thin copper sheets (**c**), for x = 2/25 in a closed (**b**) and open position (**d**).

The change in the area covered by the structure between the closed and open positions, shown in Figure 9, results in a Poisson’s ratio equal to −1. For the very thin materials used for the squares, achieving the above effect requires further adjusted axes of rotation. On the other hand, by using a very thin (0.1 mm) elastic steel sheet, one can build bendable structures that are therefore also suitable for parts that are not flat.

We observed that, in the closed position, the angle *θ* equal to the angle between the horizontal line and the edge of the square is very small and, in this case, is theoretically equal to *θ*/2 = 5.5°. In reality, it is slightly larger, taking into account the size of the rotation axis holes (approx. 1.5 mm). For a small parameter x, a large opening (large pore surface) and a relatively significant size change are obtained. In this case, it is also very easy to move from a closed position to an open position and vice versa—from an open position to a closed position. There are no compressive or tensile forces acting on the elements, only the frictional resistance due to rotation.

**Figure 10:**
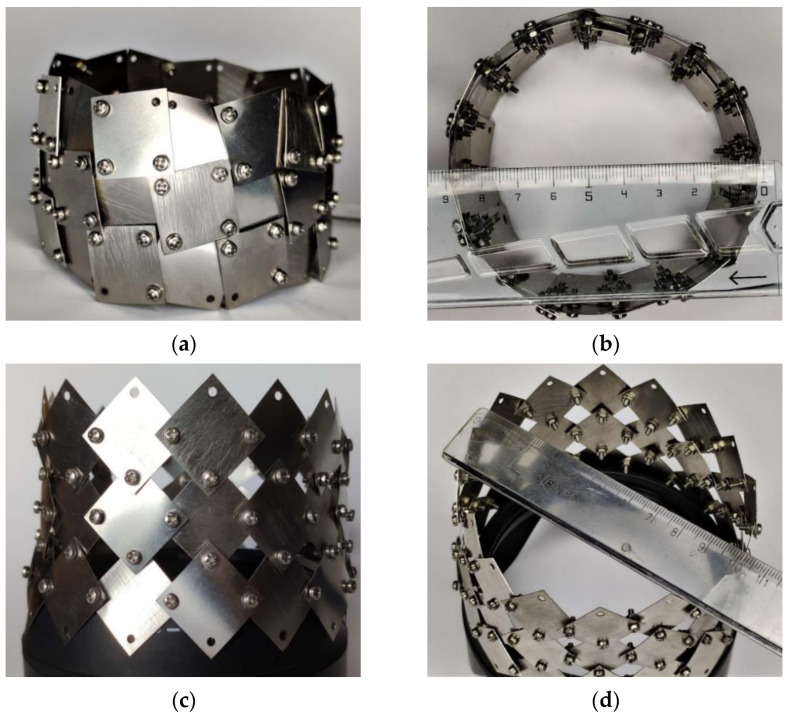
Photographs of auxetic structures in the form of a closed circle made of thin steel sheets in ‘closed’ (**a**,**b**) and ‘open’ positions (**c**,**d**).

While examining the relationships in a system of rotating squares with rotation axes on their surface, a number of models were made, including a round, closed structure made of a thin steel sheet. Figure 10 shows photographs of the some auxetic structure models created. The presented a band-like structure, which can be considered as part of a tubular structure (in which case it would require the attachment of additional square units). The thin, flexible steel sheet used for the squares might exhibit some resistance to lateral deformation. However, to confirm the existence of such a property, further technical measures for perfecting the structure are required.

The presented idea of a band with auxetic properties can be treated as an element that already achieves 3D symmetry. Although the design still lacks more precise connections on the rotation axes, it nevertheless exhibits a change of dimensions in all directions. The steel squares connected to form a kind of band can be treated as a demonstration that can be an inspiration for designing further solutions. It follows from the above that the proposed auxetic structure based on rotating squares and connected at the axes on the surface offers various possibilities for configurations, which is already known from other authors, e.g., the stent type [24].

## 4. Conclusions

In the present paper, we manufactured and studied modified auxetic structures based on rigid rotating squares. The modification consisted of the creation of a new type of connection of the squares: instead of hinges, the rotation axes located at the corners on the surface of the squares were used. The connections of the squares are free from stress since they have the features of slide bearings. The result is a stable and damage-free connection of the squares and thus a new durable auxetic structure.

It has been shown that, when the structure is stretched, as its size increases, the squares undergo both rotation and displacement. This proves that the deformation mechanism of the structure is rotational–translational in nature. Based on the mathematical analysis, the relationship between the internal theta angle and the position of the axis of rotation given by the x parameter was determined, thus, enabling the design of this modified structure. When the structure is stretched from the closed to the open position, the dimensions of the structure (*X*_1_ and *X*_2_) increase uniformly, leading to a Poisson’s ratio value of −1.

The determined Poisson’s ratio is not altered by changing the parameters, such as the size of the squares, the number of squares, and the position of the rotation axes. In the closed position, the auxetic structure is completely tight, and in the open position, it has regular pores. The size of these pores (empty spaces) is a function of the parameter x and decreases along with the increase of this parameter. The size of the pore surface can be important, for example, in adjustable filtration systems.

A number of physical models were made for the purpose of the study, two of which are shown in the present paper. Both the flat planar model and the band-shaped model made through linking the two ends of the planar model exhibited auxetic behavior consistent with the predictions of the geometric model. The simple tests that were performed indicated that such auxetic structures based on Grima and Evans’ rotational squares can be further developed and offer significant application prospects.

Considering the potential benefits of the negative Poisson’s ratio (NPR) effect in the proposed systems, one can regard this model as an encouragement for further work. Potential future ideas could involve, e.g., mechanical mesostructures allowing for mechanical control to change their dimensions.

## 5. Further Research and Applications

The geometric models created in the present report should be further validated with physical models. In the physical models, particular attention should be paid to the geometric constraints highlighted in the report. In further inquiry into systems based on rotating squares, we intend to construct multilayer auxetic materials that exhibit these properties in two dimensions. This can be applied, e.g., in expansion joints or structures in which the porosity is mechanically altered, such as mesoscale structures.

## Data Availability

Data have contained within the article.
